# Variation in Honey Bee Gut Microbial Diversity Affected by Ontogenetic Stage, Age and Geographic Location

**DOI:** 10.1371/journal.pone.0118707

**Published:** 2015-03-13

**Authors:** Zuzana Hroncova, Jaroslav Havlik, Jiri Killer, Ivo Doskocil, Jan Tyl, Martin Kamler, Dalibor Titera, Josef Hakl, Jakub Mrazek, Vera Bunesova, Vojtech Rada

**Affiliations:** 1 Department of Microbiology, Nutrition and Dietetics, Faculty of Agrobiology, Food and Natural Resources, Czech University of Life Sciences Prague, Prague, Czech Republic; 2 Institute of Animal Physiology and Genetics v.v.i., Academy of Sciences of the Czech Republic, Prague, Czech Republic; 3 Bee Research Institute at Dol, Libcice nad Vltavou, Czech Republic; 4 Department of Zoology and Fisheries, Faculty of Agrobiology, Food and Natural Resources, Czech University of Life Sciences Prague, Prague, Czech Republic; 5 Department of Forage Crops and Grassland Management, Faculty of Agrobiology, Food and Natural Resources, Czech University of Life Sciences Prague, Prague, Czech Republic; Fish Vet Group, THAILAND

## Abstract

Social honey bees, *Apis mellifera*, host a set of distinct microbiota, which is similar across the continents and various honey bee species. Some of these bacteria, such as lactobacilli, have been linked to immunity and defence against pathogens. Pathogen defence is crucial, particularly in larval stages, as many pathogens affect the brood. However, information on larval microbiota is conflicting.

Seven developmental stages and drones were sampled from 3 colonies at each of the 4 geographic locations of *A. mellifera carnica*, and the samples were maintained separately for analysis. We analysed the variation and abundance of important bacterial groups and taxa in the collected bees.

Major bacterial groups were evaluated over the entire life of honey bee individuals, where digestive tracts of same aged bees were sampled in the course of time. The results showed that the microbial tract of 6-day-old 5th instar larvae were nearly equally rich in total microbial counts per total digestive tract weight as foraging bees, showing a high percentage of various lactobacilli (Firmicutes) and *Gilliamella apicola* (Gammaproteobacteria 1). However, during pupation, microbial counts were significantly reduced but recovered quickly by 6 days post-emergence. Between emergence and day 6, imago reached the highest counts of Firmicutes and Gammaproteobacteria, which then gradually declined with bee age. Redundancy analysis conducted using denaturing gradient gel electrophoresis identified bacterial species that were characteristic of each developmental stage.

The results suggest that 3-day 4th instar larvae contain low microbial counts that increase 2-fold by day 6 and then decrease during pupation. Microbial succession of the imago begins soon after emergence. We found that bacterial counts do not show only yearly cycles within a colony, but vary on the individual level. Sampling and pooling adult bees or 6th day larvae may lead to high errors and variability, as both of these stages may be undergoing dynamic succession.

## Introduction

Honey bees are a key species for agriculture, contributing significantly to the human food supply [1]. Recent losses of *Apis mellifera* and the potential association of these declines with various infectious agents highlight the need for an increased understanding of innate bee immunity and mechanisms that help them adapt to environmental stress. One of these factors may be their gut microbiota; however, little is known regarding the role of beneficial microbes in honey bee health [2,3].

Social insects provide unique resources for studying microbial symbionts because of the high density of individuals within colonies, sharing of food and other resources and the coexistence of colony members from multiple generations [4]. These bacterial communities vary immensely in total size, composition, location and functions within the individual parts of the gut [5]. The adult honey bee hosts up to 10^9^ bacterial cells, consisting of 8 abundant phylotypes making up to 95% of the total bacteria that appear to be specific to social bees [6]. The maintenance of this stable and distinct microbial community depends on the nutrition and social lifestyle of these insects [7,8], environment [8,9] and ontogenetic stage [5,10,11]. This dynamic system has also been shown to follow seasonal trends [12,13]. Numerous studies have been conducted to characterize adult honey bee diversity using new cultivation-independent techniques, but fewer studies have examined the larvae. The microbiome of honey bee larvae can be highly variable, and particularly older, culture-based studies have not revealed any highly specific microbial patterns [14]. However, later PCR-based methods in the larval and adult intestine and rectum revealed a few classes of Gammaproteobacteria, recently identified as *Gilliamella apicola* and *Frischella perrara* [11,15] as well as a presence of Betaproteobacteria (*Snodgrassella alvi*) [5,7,12,15–18] and Acetobacteria [19] species in the larval gut. The presence of members of the genus *Lactobacillus* appears to be rather random; however in larval stages were detected [11,20]. Unlike many other insects, honey bee larvae defecate only shortly before pupation, making it difficult to control the microbial environment [11]. Specific knowledge regarding the dynamics and variation in the larval gut microbiome is very important, as larvae are considered to be a focus for probiotic applications and in aiding defence against pathogens and colony health [4,10]. Probiotic bacteria are known to be promoters of host body defence by triggering a humoral immune response and creating an intestinal immunological barrier [4,21,22]. As such, many studies examining bee microbiota were related to defence against the major pathogen *Paenibacillus larvae*, the causative agent of American foulbrood [10].

Several bacterial strains or a mixture of strains are thought to be beneficial for honey bees and have been considered for probiotic supplementation, mainly of the *Lactobacillus* and *Bifidobacterium* spp.; however, the assumption that lactobacilli or bifidobacteria have beneficial effects in honey bees may be oversimplified as the physiological dynamics in the bee gut microbiome are not well-understood. Between 3 and 4 distinct classes of lactobacilli have been identified and were recently characterised: *Lactobacillus apinorum* [23], which is phylogenetically similar to *Lactobacillus kunkeei*; Firmicutes (Firm) 4 clade represented by *Lactobacillus mellifer* and *Lactobacillus mellis* and the Firm 5 clade represented by recently identified phylogenetically close species of *Lactobacillus melliventris*, *Lactobacillus kimbladii*, *Lactobacillus helsingborgensis*, *Lactobacillus kullabergensis* [23] and *Lactobacillus apis* [24]. Bifidobacteria are present in relatively low abundance in honey bees. Higher variability of bifidobacteria was recently observed in closely related bumblebees [25–27]. However, the role of Gamma (Gammaproteobacteria represented by the strains *G*. *apicola* or *F*. *perrara*) [15,28] as a large and honey bee-specific microbial group remains unexplored, although they may also take part in pathogens defence [29].

Previous studies of bacterial populations as honey bee gut symbionts have not examined the large variation between geographical locations or individuals. Although differences in microbiota were seen between honeybees from different parts of the world [30] nothing is known about differences in bacterial populations of relatively close apiary sites with slightly different environments. Information on such interactions and inter-and intra-colony microbial variation is lacking, particularly for larval and pupal stages. The aim of this study was to compare the microbial populations in 7 developmental stages (+ drones) in honey bee colonies living within same location and within different apiary sites. We searched for patterns and variability in the honey bee microbiome, focusing on selected *Gilliamella* and lactobacilli strains using 16S rRNA denaturing gradient gel electrophoresis (DGGE) and quantitative real-time (qRT)-PCR. We examined whether honey bee microbial populations are affected by location and thus food sources of microbial inoculation. Our goal also was to gain insight into microbial dynamics during honey bee development.

## Materials and Methods

### General experimental approach

Two experiments were performed. The experiment referred to as EXP1 involved sampling of various developmental stages from 4 different locations and was used for DGGE analysis and follow-up statistical evaluation. The other referred to as EXP2 was conducted in a single hive, tracking microbial populations of bees of similar oviposition time and was also referred to as a “single bee” model.

### Honey bee samples

In EXP1, we compared microbiota in various developmental stages on 4 geographical locations and samples from total 12 colonies of *A*. *mellifera carnica* were collected from the following locations: Dol (50°12'23.9"N 14°21'58.8"E), Ustrasice (49°20'35.6"N 14°40'55.2"E), Postrizin (50°13'34.0"N 14°22'42.5"E) and Hostice (50°12'23.3"N 14°24'10.8"E) (S4 Fig.). Hereby, we certify that the samples were collected on either private land (land of Bee Research Institute Dol) or land commercially rented, we solely hold responsibility for ethical approaches and can be contacted later for confirmation. The field studies did not involve endangered or protected species. EXP2 samples originated from one hive in the Dol location. Sampling was conducted on 31 July 2012 (EXP1), and continued for 60 days for the time-course experiment (EXP2).

For EXP1, 3 hives were randomly selected at each location and from each hive, samples of 1-, 3- and 6-day-old larvae, white and black pupae, young bees and foraging bees and drones were collected. One-day-old larval sample corresponded to the 1^st^ instar larva shortly after breaking the egg chorion and forming the C-shaped position. The 6^th^-day-old larvae corresponded to the 5^th^ instar, with the gut completely filled with a yellowish material and corresponding to the last feeding stage LF3 [31,32] prior to sealing. White pupae were collected with red brown to dark brown eyes and no signs of body pigmentation (Pr–Pd) [32]. Black pupae were acquired when they showed medium thorax pigmentation. Young bees and drones were bees randomly collected after emergence from the combs and nectar foraging bees were sampled from the landing board. Approximately 5–10 individuals were collected in disposable tubes and frozen immediately on dry ice. Bee management and samples represented traditional beekeeping practices in the Czech Republic.

For EXP2, to obtain controlled oviposition and larvae of the same age, the queen was secured to a broodless comb in a colony using an excluder cage for a 4-h period. Next, the brood was left to develop and was sampled on days 2, 5, 8, 12, 16 etc. Each newly emerged bee was labelled with paint and later, only marked bees were sampled. Each sample was a pooled sample of 3–10 individuals. A total of 16 samplings were conducted during this period.

Entire tube-like digestive tracts (crop, midgut, ileum and rectum) were removed from each honey bee stage, pooled and weighed. Exceptions included samples of 1-day larvae which were swabbed from 10 honeycomb cells by cotton swab, submerged in 200 μL sterilized water and centrifuged within 90 sec to 9000 rpm. Next, the pellet was used for isolation of total bacterial DNA. Three-day-old whole larvae were homogenised. For other samples, approximately 50–200 mg of pooled digestive tracts were used for isolation of total bacterial DNA using the ZR Faecal DNA MiniPrep kit (Zymo Research, Irvine, CA, USA).

### Denaturing gradient gel electrophoresis and sequencing

Amplification of the total bacterial community was conducted by targeting 200 bp partial 16S rRNA gene sequences with the universal bacterial primers 338GC and RP534 under previously described conditions [9]. PCR products were analysed on a DGGE gel (gradient from 35–65%) according to the method of Mrazek et al. [9].

Appropriate standards containing a mixture of PCR products of 5 known microorganisms were loaded in the centre of gels defined to minimize gel variability and used for multi-gel comparison was conducted using BioNumerics 6.6 software (Applied Maths, Sint-Martens-Latem, Belgium). Lanes were manually aligned and band positions were identified from corrected intensity plots. Band matching was accomplished by using the following BioNumerics settings: 7% minimum profiling, 0% gray zone, 0% minimum area, and 0 shoulder sensitivity. Comparison between samples loaded on different DGGE gels (S1 Fig.) was completed using normalized values derived from known standards (used as external references). A matrix of the relative band intensity values of 17 major identified bands was prepared for all gel lanes (S1 Table).

Two to three DNA bands with the same normalised Rf values were cut out of the polyacrylamide gel using a sterile scalpel blade to confirm their correct alignment (selected bands shown in S1 Fig.). Bacterial species were detected using the primers FP341 and RP534. The bands of interest were sequenced on a 3100 Avant Genetic Analyser (Applied Biosystems, Foster City, CA, USA) in the Centre of DNA Sequencing (Institute of Microbiology of the ASCR, v. v. i.). The resulting sequences were compared with the GenBank database using the BLAST algorithm. Eukaryotic DNA bands were omitted for profiling. Some bands showed the presence of heterogeneous DNA, but were used for statistical analysis. The sequencing revealed that 2 of the single 16S rRNA sequences generally appeared as multiple bands at 2 Rf values, likely because of variation between the GC-clamp primers, which was previously described by Rettedal et al. [33]. For multivariate statistical analysis, these were considered as different bands but were pooled for heatmap construction. Some of the bands of low abundance or from the parts where matching may be of low precision were omitted.

Three of the lactobacilli, *G*. *apicola* and *F*. *perrara* strains used as standards for the DGGE profiling were isolated from adult honey bee digestive tracts. Their 16S rRNA gene sequences and those of honey bees related to Firm, or selected strains of the family Orbaceae obtained from the National Center for Biotechnology Information database (www.ncbi.nlm.nih.gov), were aligned using ClustalW within the MEGA 5.05 software [34]. Alignments provided by the ClustalW algorithm were improved by removing hypervariable positions using the program Gblocks [35]. Evolutionary distance matrices were generated using the Cantor & Juke model. Two phylogenetic trees for both lactobacilli (see S2 Fig.) and *G*. *apicola/F*. *perrara* were constructed (see S3 Fig.).

### Real-time PCR analysis

Quantification of bacterial DNA was conducted using mx3005P thermocycler (Stratagene, La Jolla, CA, USA) with Gammaproteobacteria (1080γF, γ1202R), Bacteroidetes (798cfbF, cfb967R), Firmicutes (928F-Firm, 1040FirmR) and Actinobacteria (Act920F3, Act 1200R) [36].

### Statistical analysis

To examine the relationship between colony location and honey bee development stage on the total distribution of major abundant bacterial strains, we performed redundancy analysis (RDA) on all data using the CANOCO 4.5 program (Microcomputer Power, Ithaca, NY, USA) [37]. RDA was preferred over principal component analysis because of the advantages of direct association of constrained canonical axes with groups of independent variables. CANOCO provides an advantage of separate centring and/or standardization within response variables (bacterial strain abundance) and within samples (each combination of ontogenetic stage, location and replicate). To test our hypothesis, 2 models were arranged where both included the abundance of bacterial strains as response variables and location-to-ontogenetic phase interaction as independent variables. When absolute values of strain abundance were investigated, only the centring (results in zero average) of each strain were used. If only the proportions of strain abundance was considered, the centring of each strain as well as the centring and standardizing (results in norm equal to one) of samples were used.

The statistical significance of first and all other constrained canonical axes was determined using the Monte Carlo permutation test (499 permutations). An ordination diagram was created in CanoDraw for graphical visualization of the results. Heatmaps were constructed in MS Excel (Microsoft Corporation, Redmond, WA, USA). For analysis and visualisation of qRT-PCR data, we used IBM SPSS Statistics ver. 20 (IBM, Armonk, NY, USA).

## Results

### DGGE fingerprinting patterns

**Fig 1:**
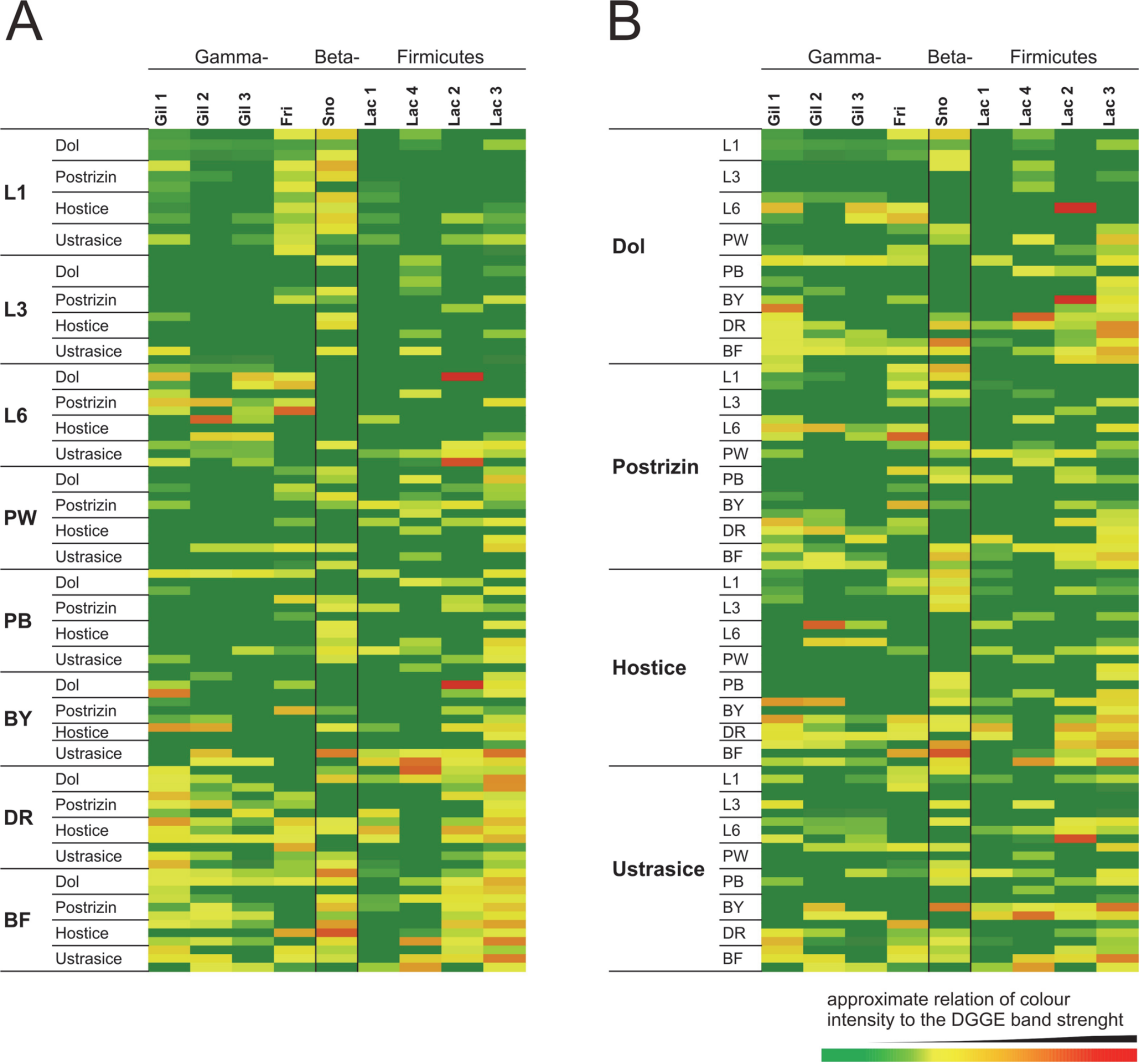
Heatmap summarising the relative density of dominant denaturing electrophoresis bands of the 16S rRNA amplicon profiles of the total gastrointestinal tract contents of several honey bee ontogenetic stages. 1-, 3- and 6-day old larvae (L1, L3 and L6, respectively), white and black pupae (PW, PB), young bees, drones and flying bees (BY, DR and BF, respectively) collected in 4 different locations (Dol, Postrizin, Ustrasice, Hostice). Samples are sorted by ontogenetic stage (A) and location (B). The colours refer to relative band strength according to the colour key.

Samples of honey bees in various ontogenetic stages were acquired from 4 apiary sites within 10–100 km distance of each other (S4 Fig.), and 3 colonies from each apiary site were investigated to determine bacterial diversity. This experiment is referred to as EXP1. DGGE fingerprinting profiles of 200-base pair (bp) amplicons of the 16S rRNA V3 region showed between 20–30 bands. Of these, the 17 abundant or well aligning bands (S5 Fig.) were matched manually through multi-gel alignment of all 96 samples. Selected major band intensities were displayed in a heatmap (Fig. 1). This visualisation revealed that all three *G*. *apicola* (Gamma1) strains occurred rather randomly and at very low intensities in 1^st^ instar larvae (L1) and at even lower intensities in 4^th^ larval instar (L3), but became abundant bands in 5^th^ instar larvae prior capping and defecation (L6). In later stages, they were mostly absent in both pupal stages (white pupa, PW, and black pupa, PB) and began occurring randomly in samples of young bees (BY) in some apiary sites. These strains were very abundant in foraging bees (BF) and drones (DR) with similar distribution profiles. At each apiary site, there were colonies with absent or highly abundant *Gilliamella* sp. (Gil) strains. Gil 1 (100% similarity to wkB1^T^) and Gil 2 was generally present as a more intense band than Gil 3. *Frischella perrara* (Fri) (Gamma-2) showed a very similar pattern to *Gilliamella* sp. strains (Fig. 1).

Betaproteobacterium *Snodgrassella alvi* (Sno), a typical and recently characterised honey bee host-specific microorganism, showed different distribution patterns from that of Gamma. Sno was nearly absent in L6 samples with an apparent exception: 1 colony from the Ustrasice site, where it also appeared in a higher abundance in both PW and PB. Interestingly, this colony in Ustrasice showed clinical signs of sacbrood virus infection, with larvae changing in colour and their mouth parts turning black. However, no general conclusion could be drawn from this case. *Snodgrassella alvi* appeared to occur with higher band intensities in PB samples than Gamma. In foraging bees, this bacterium was ubiquitous.

In the heatmap (Fig. 1), band intensities of the 4 most abundant and clear bands corresponding to *Lactobacillus* spp. strains are shown. All 4 selected lactobacilli strains clearly appeared to have higher band intensities in later ontogenetic stages. Lac 3 (100% similarity to *L*. *apis* R4B^T^ [24] and 99% to Hma 11 [23]) was clearly more abundant than the others, followed by Lac 2 (100% similarity to *L*. *kimblandii* Hma2N^T^ [23]). Lac 2 generally occurred randomly as a very intense band twice in different hives in the Dol apiary. In contrast to Lac 3, presence of Lac 2 was much rarer and was mostly absent in pupal or larval stages, with the exception of the Postrizin location where this strain appeared to be more abundant in pupae rather compared to Lac 3. The Lac 1 strain (99% similarity to *L*. *helsingborgensis* Bma5N^T^ [23]) showed lower and rather patchy distribution in all stages.

Some bands, such as Gil 2, Fri, Lac 2 and Lac 3, appeared to be more typical for adult stages, while Sno, Lac 3 and Lac 4 (98% similarity *to L*. *melliventris* Hma8N^T^ [23]) appeared more frequently in pupae. In contrast to our expectations, no typical patterns of these selected strains were observed to be characteristic for one particular location.

### Redundancy analysis of DGGE fingerprints

Microbial diversity based on DGGE profiles was statistically evaluated using redundancy analysis. A matrix of relative band intensities was used after grouping results from 3 colonies at each apiary site for simplification.

When the absolute abundance of strains was examined, the apiary site-to-ontogenetic stage interaction was significant (*P* = 0.002) and explained 43% of strain variability (all canonical axes), which was nearly 2-fold higher than when the 2 factors were evaluated separately (data not shown). Approximately half of the variability in total abundance of microbial strains may contribute to the difference among the 7 major ontogenetic categories and 4 apiary sites.

**Fig 2:**
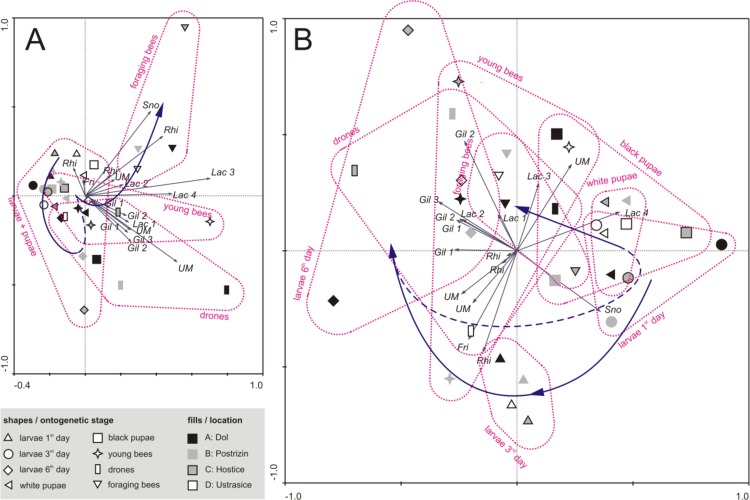
Biplot from redundancy analysis (RDA) explaining the distribution of honey bee ontogenetic stages according to major bacterial strain abundance. Test of interactions between factors location and ontogenetic stage: A, crude data considering absolute DGGE band intensities; B, centred and standardized data considering relative band intensities. Abbreviations: Gil, *Gilliamella apicola*; Sno, *Snodgrassella alvi*; Lac, *Lactobacillus* sp.; Rhi, *Rhizobiales* bacterium; Fri, *Frischella perrara*, UM, unknown multiple—probable DNA heterodimer. For further strain descriptions, see S5 Fig. Dotted shapes surrounding each ontogenetic stage were created as an aid in visualization. Eighteen bacterial strains occurring as major 16S rDNA DGGE bands were used for statistical analysis, while only selected strains are plotted as arrows. Same descriptions are for bands of the same sequence occurring at multiple locations of the line. Blue arrows show hypothetical developmental timeline. Its dotted part is ambiguous.

In Fig. 2A, the first canonical axis (horizontal) explained 15.3% (i.e. 35% of total explained) of strain variability and separated stages according to age with L1, L3, PW and PB on the left, L6 in the middle of the plot and adult stages mainly on the right side of the plot. The abundance of nearly all strains clearly increased with age (towards the right side). The highest variability according to locality was observed within drones and young bees, where the total strain abundance of these stages may be more similar to adults (e.g. drones in Dol) or larval stages (e.g. drones in Ustrasice). The data distribution for the second canonical axes (vertical) showed a trend in the differences within adult stages with drones in the lower plot, young bees in the middle and foraging bees in the upper part of the plot. *Lactobacillus* Lac 3, Lac 4, *Snodgrassella alvi* (Betaproteobacteria-Beta) and unknown *Rhizobiales* bacterium (Rhi) (Alphaproteobacteria-Alpha) appeared to be strains that were generally characteristic for foraging bees, while *Gilliamella* strains were characteristic for the microbiota of young bees, particularly drones. Although the samples were pooled, young bees at some locations appear to be less populated by distinct microbiota and are thus appear on the left side of the first canonical axis with larvae and pupae. This might be to the fact, that microbial successions in young bees may be faster at some sites or colonies compared to others.

The highest variability was observed within young bees and drones in the direction of first axis, in contrast to 6^th^-day larvae and foraging bees in the direction of the second axis. This variation may have resulted from our experimental design, varying age of the individuals, time since the emergence and phase of microbial succession.

When the relative proportion of strain abundance was evaluated using redundancy analysis (RDA), the combination of location and ontogenetic stage was also significant (*P* = 0.002) and explained 34% of strain variability (all canonical axes). In Fig. 2B, the first canonical axis explained 6.2% of variability and clearly distinguished 6^th^-day-old larvae in the left area from other larval or pupal stages on the right, showing higher proportion of *Gilliamella* strains on the left, compared to higher proportions of *Lactobacillus* Lac 4 and *Snodgrassella alvi* on the right. The second axis suggests a specific proportion of microbiota of L3 larvae (lower part) with a higher proportion of unknown *Rhizobiales* bacterium and *F*. *perrara* in contrast to most of the L6 larvae, drones and young bees with a higher proportion of *G*. *apicola* Gil 1–Gil 3 and Lac 3. Similarly to total abundance, proportions of strains were strongly affected by the apiary site. Variability was mainly in the direction of the second axis for drones, young bees and partially L6 larvae. A limitation of DGGE studies may be that some bands were not examined, as they occurred at rather low intensities and correct matching would be difficult.

### Quantitative PCR of microbial populations between apiaries

Real-time qPCR was conducted to quantify bacterial abundances using primers specific to major microbial populations, including Firmicutes, Gammaproteobacteria and minor groups of Actinobacteria and Bacteroidetes. Although bacterial counts varied highly among samples of the same stage, several patterns were recognized. During early development until day 3, in more than 90% of colonies, Gammaproteobacteria (Gamma = G) prevailed over Firmicutes (Firm = F) (G: 5.6 × 10^6^ vs. F: 1.4 × 10^5^; the means of gene copies per gram of digestive tract content), whereas when the 5^th^ instar larva (L6) was prior to sealing, the ratio changed and Firm dominated over Gamma in 83% of hives (G: 1.9 × 10^7^ vs. F: 2.5 × 10^8^). After larval defecation and during pupation, bacterial counts decreased significantly; however, the dominating bacterial group was again Gamma in 80% of hives (G: 8.5 × 10^6^ vs. F: 7.2 × 10^5^) and was the major component in 60% of pigmented pupae (G: 6.8 × 10^7^ vs. F: 1.5 × 10^6^). Among newly emerged bees collected randomly from the comb, 55% contained higher counts of Firm (G: 1.7 × 10^7^ vs. F: 2.3 × 10^8^), and this ratio was further increased in drones and flying bees where Firm dominated in approximately 90% of samples (G: 1.3 × 10^7^ vs. F: 3.1 × 10^8^ and G: 5.1 × 10^7^ vs. F: 5.4 × 10^8^).

Moreover, the Bacteroidetes group and Actinobacteria were analysed using qRT-PCR. These groups were much less abundant; however, they appeared to follow a similar pattern. Variation between samples was high. In this study, we did not focus on 2 other microbial taxa present in the honey bee population: Alpha and Betaproteobacteria.

### Changes in bacterial populations during the life span of a “single” honey bee

**Fig 3:**
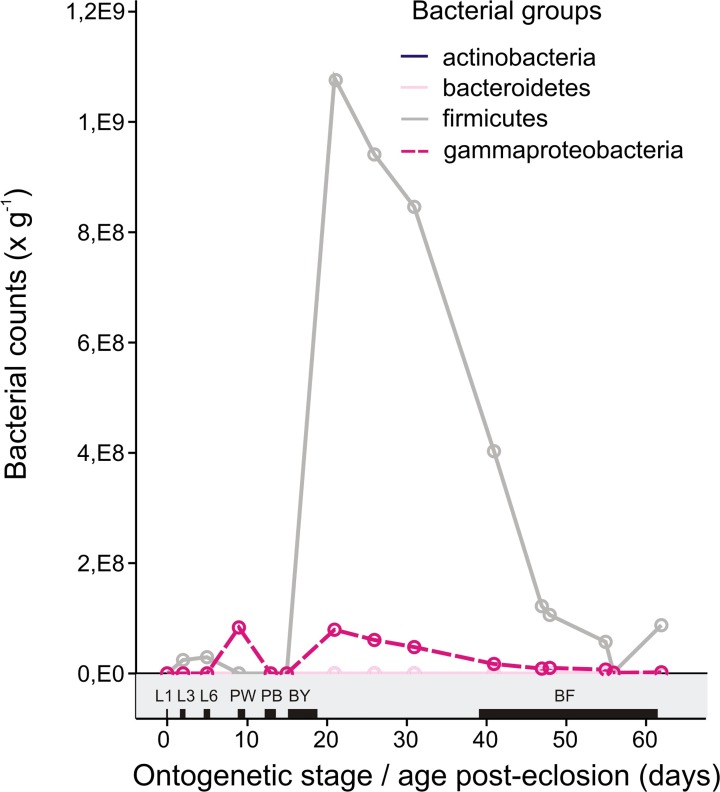
Dynamics of selected bacterial groups in the total gastrointestinal tract during development and aging of a “single” honey bee. Data were obtained by collecting pooled samples of sister honey bees from the eggs of the same oviposition. Young bees were marked by paint shortly after emergence. Legend in the grey field provides a link to the first experiment EXP2 described here and shows at which approximate time points the samples for EXP1 were collected.

Another experiment (EXP2) was conducted to gain insight into the variation and dynamics of bacterial population changes during honey bee ontogenesis in one selected hive. The results showed that 4^th^ and 5^th^ larval instars were dominant in Firmicutes (Fig. 3); although the counts (gene copies per gram of digestive tract content) were generally low (2.9 × 10^7^ in L6).

**Fig 4:**
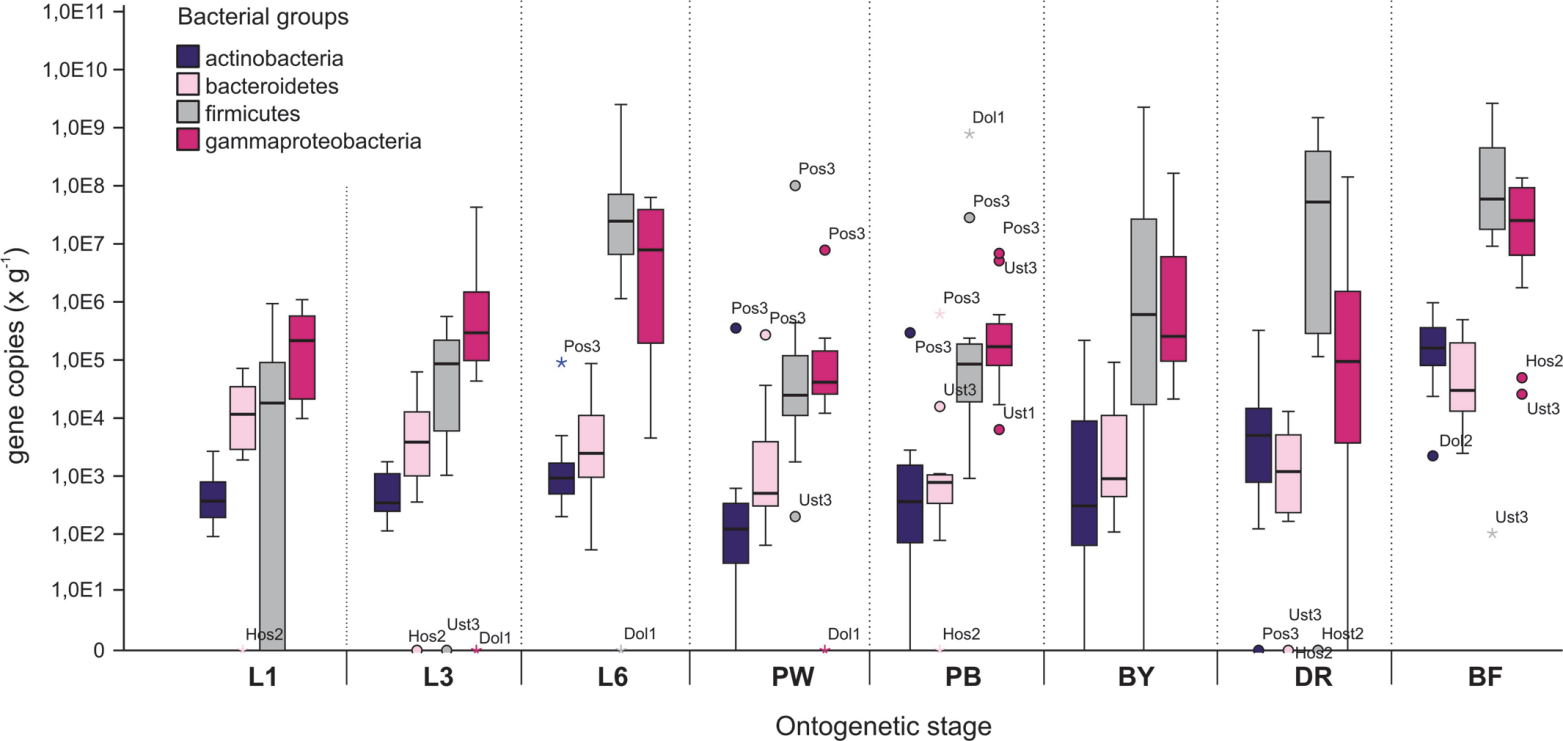
Boxplot of quantitative real-time PCR (qRT-PCR) data of the abundance of selected bacterial groups in pooled samples of total gastrointestinal tract of each honey bee ontogenetic stage. The Y axis shows log-transformed copies of the 16S rRNA gene per gram of honey bee gastrointestinal tract. Boxes show pooled data from 4 locations and 3 hives at each location. 1-, 3- and 6-day old larvae (L1, L3 and L6, respectively), white and black pupae (PW, PB), young bees, drones and flying bees (BY, DR and BF, respectively). The codes of the outliers refer to the location (Pos: Postrizin, Hos: Hostice, Ust: Ustrasice) and colony number (1, 2, 3).

After defecation and capping, their counts decreased to (3 × 10^2^). During pupation, an interesting rise in Gamma abundance was observed in white pupae samples (8.3 × 10^7^), which was in this case an order of magnitude higher than the mean of the counts observed in EXP1 and rather similar to its maximum value. In black pupae, these bacteria were suppressed and newly emerged bees continued to show very low bacterial counts. The newly emerging bees appeared to be inoculated and bacterial counts of both Gamma and Firm groups within 6 days post-emergence quickly increased to the highest bacterial counts (G:7.9 × 10^7^ vs. F: 1.8 × 10^9^). Shortly after this rapid inoculation, both bacterial groups began decreasing in abundance, primarily in a linear manner as the honey bee aged (Fig. 4). Between 17–24 days after emergence, honey bees become foragers under normal circumstances [38]; both counts of Firm and Gamma were reduced by half compared to young bees at 6 days postemergence. At day 40, counts of Firm were as low as 4 × 10^8^. It is clear that the sampling of young bees of unspecified age for microbiological analyses is linked with large variation error as the bees were in the process of inoculation and rapid succession.

## Discussion

### Microbiota of 1^st^ instar larvae

Our study revealed several interesting insights into the dynamics of honey bee microbial communities. First instar larvae generally showed very low bacterial counts, which is in agreement with previous studies [3,39–41].

Whereas the Gamma group was slightly more abundant in this ontogenetic stage with 3.3 × 10^5^ gene copies/g, the counts of Firm were 1.3 × 10^5^ copies/g. This appears to be in contrast with the generally accepted notion that Gamma are only minimally present during in this early stage. However, Mohr and Tebbe [11] previously found Gamma OTU in honey bee larvae and thus, their presence even in later larval instars cannot be ruled out. Our primers did not specifically distinguish between the core microbial set including *Gilliamella* and *Frischella* strains and other bee-non-specific Gamma. In this early ontogenetic stage, royal jelly proteins are thought to suppress microbial growth [3]. Earlier studies have suggested that the presence of microbes in the larval stage digestive tract was due to unwanted contamination [41]. Although our amplicon-based study revealed numerous copies of the 4 bacterial groups examined and the occurrence of bands characteristic of core honey bee microbiota in the DGGE profiles in our study suggest their presence in this developmental stage, the results might be biased by the fact that we used a cell content smear rather than an isolated larval tract.

### Microbiota of 2–4^th^ instar larvae

As the larva ages towards the 4^th^ instar on day 3 of larval development, Alpha and Betaproteobacteria represented by an unknown *Rhizobiales* strain, *S*. *alvi* and, rarely in some of the samples, *F*. *perrara* remained among the frequently observed DGGE bands (Figs. 1 and 2B). Fingerprinting did not reveal any other typical bands, only the very random occurrence of the Firm and Gamma groups and low intensities of lactobacilli. The qRT-PCR method, however, showed that Gamma counts increased to 5.5 × 10^6^ gene copies/g of the entire tract, whereas Firm remained at approximately the same level (1.5 × 10^5^) (Fig. 3). This is clear even from evaluation of the DGGE patterns (Fig. 1). Box plot values in Fig. 3 may appear lower than the counts mentioned, as they do not show mean but median values after elimination of outliners.

### Microbiota of 5^th^ instar larvae

Major changes were observed in 5^th^ instar larvae collected shortly after the last feeding. The Firm group averaged 2.5 × 10^8^ and Gamma 1.9 10^7^ gene copies per gram of digestive tract content. DGGE fingerprinting revealed a decrease in *S*. *alvi* and increase in the diversity of all Lac and Gil strains. RDA (Fig. 2B), showed very high variation among these L6 samples, particularly varying Gil strain abundance. Previously, *S*. *alvi* was found to be an important factor in biofilm formation, forming a bottom layer directly associated with the epithelial tissue, followed by the thick layer of Gil. Layered *Snodgrassella*/*Gilliamella* biofilm may function as a pathogen barrier as observed in *Bombus* spp. larval samples [29]. It is possible that the reduction of *S*. *alvi* during ontogenesis is related to morphological changes, during which the gut intima is shed [42]; between locations, it may be affected by developmental speed in each colony.

The abundances of Firm and Gamma in larval and pupal instars were not fully supported by the EXP2 time-course experiment. The results of these experiments are shown in Fig. 3. The abundances of Gamma and Firm were lower during the larval development stages, which may be because of the natural variance between samples and because many of the strains occur at sporadic abundance; this is consistent with the hypothesis of a disease state. EXP1 and EXP2 results were difficult to compare because of their different designs.

### Microbiome of young bees, drones and flyer bees

After pupation, the counts of both Firm and Gil decreased by nearly 2 orders in magnitude to means of 8.5 × 10^6^ and 7.2 × 10^5^ gene copies/g, after which growth continued slowly (Fig. 3). With the exception of one sample, RDA (Fig. 2B) distinguished black and white pupae from the L6 larvae based on the higher abundance of either *L*. *apis* or *L*. *melliventris*, whereas *L*. *apis* was more typical for black pupae and young bees. New bees are quickly inoculated within a maximum of 6 days post-emergence and showed mean values of 2.3 × 10^8^ gene copies of Firm and 1.8 × 10^7^ of Gamma, counts which were very similar to those observed in 5^th^ instar larvae L6. This has recently been confirmed by Powel et al., who found that the maximum is reached depending on hive in 16 days [43].

The profile and bacterial counts of drones and young bees were very similar (Figs. 1 and 3) with drones showing only slightly lower counts of Gamma and higher counts of Firm. RDA revealed that adult groups were well-separated according to strain abundance along the second axis in Fig. 2A, indicating some characteristic differences in strains distribution among stages.

Comparing to young bees, the number of gene copies of Firm and Gamma in pooled foraging bees, was significantly higher: 5.4 × 10^8^ and 5.1 × 10^7^, respectively.

### Role of location in microbial composition

RDA revealed that despite clear drift in strain proportion from larval stages back to pupal stages and to adults, groups of bees and drones highly varied with location (Fig. 2B), showing some visible similarities of stages within a location (e.g. drones, young bees and L6 from Hostice in upper left side of the Fig.). The relative frequencies of core phylotypes are known to vary considerably among individuals in the same hive; in many cases, bacteria identified as core to the gut were not consistently found in foraging bees [12], and the same may apply for L6 larvae. However, based on the heat map, no characteristic pattern was observed for any of the locations. At each location, all 3 hives appeared to be very different from each other and, rather than location, simple variability between hives was important.

### Seasonal rhythms and ontogenetic changes in microbial profiles

Some studies have provided insight into seasonal time-course changes in honey bee microbiota; however these results are still very fragmented and studies have not examined complexity. A study by Ludvigsen [13] clearly showed that while *G*. *apicola* follows seasonal changes and its counts continually decrease during the year, with the lowest values observed in October bees, while *S*. *alvi* counts rise during that period. Some periods of the year show higher variation among individuals in a single colony and colonies. Similarly, recent studies have shown a reduction in Firm-5 lactobacilli from spring to the fall by approximately 25% in foraging bees [12]. Studies on honey bee microbiota conducted at a particular time point may be affected by these seasonal rhythms. High variability may also be observed in different years. Mohr and Tebbe observed significant differences in microbial profiles from one year to another [11]. This information is very important, as it may help in the design of proper probiotic supplementation strategies with respect to physiological conditions in the honey bee gut. We found that new bees are inoculated within a maximum of 6 days post-emergence; after that, bacterial counts decrease gradually over their lifetimes. Recent study of the Yale University team [43] came to similar time frame of six to eight days post-emergence needed for the honey bee microbiome to reach maximum communities’ richness and abundance, however, our study goes further by following the bees for their entire life.

### Controversies in larval microbiota richness and composition

Colonisation of the larval gut is thought to start with strains of *L*. *kunkeei* [39] occupying the niche, dominating *Acetobacter* spp. (Alpha 2.2) abundance in early instars, and may be related to changes in the diet, which now contains pollen. Many pollen-associated bacteria species are considered to be contaminants, not belonging to the honey bee core-microbiota [3]. Older culture-based studies generally found zero-to-low microbial abundance in larvae [14,41], but may be biased by the method and sometimes show contrasting conclusions, particularly for PCR-based methods. However, even with the widespread use of PCR-based methods, information regarding larval microbiota remains inconsistent, with many opposing theories. Few studies have examined this topic, however, they have not examined variability, which may have a large impact; these studies also sampled only a few individuals of *A*. *mellifera* or used pool samples from 1 colony [5,11,20]. Moreover, is not clear how management practices, breeding lines or colony location shape the microbial population. A study by Vojvodic [40] found large differences between managed *A*. *mellifera* and unmanaged Africanized bees patterns of larval microbial succession. Larval stages of Africanized bees were much more diverse in microbial species richness, in contrast with previous studies contained nearly 50% Firm-4 and Firm-5 [40]. Their conclusions were based on pooled samples from 3 colonies. In agreement with our results, a study of Vojvodic [40] confirmed that 4^th^ and 5^th^ instar larvae are already populated by Firm-5 and Firm-4 strains, although Gammaproteobacteria were not found. Gammaproteobacteria such as *Gilliamella* spp. have, however, been reported as part of the larval microbiota earlier [11,44].

An important limitation and source of discrepancies between recent studies focused on honey bee larvae microbiota may be that authors do not use a clear classification of honey bee preimaginal stages. As larval succession is very dynamic, similarly to physiological processes during morphogenesis, these studies require proper descriptive criteria. Thus, we propose using strict classification described by previous studies [31,32] for *A*. *mellifera carnica* and Africanized bees, respectively.

In our study, larval samples of later instars in some colonies were essentially free of *Lactobacillus* strains with sporadic and quite low abundances, but nearly all samples showed bands of *F*. *perrara*, *S*. *alvi* and *G*. *apicola* Gil 1 (Fig. 1). The results obtained by DGGE profiling should be interpreted with caution because although all bands at the same Rf were matched with the highest thoroughness and each line was checked from 2–3 randomly sequenced bands, errors may occur and this data should be examined using statistical methods.

## Conclusions

Active microbiota in honey bees differs in species richness and total abundances across the ontogenetic stage of honey bee and hive location. There were no clear patterns visible between different geographic locations; however, using DGGE and RDA, we identified several strains of *Lactobacillus* and *Gilliamella* spp. with characteristically higher but patchy abundance in various developmental stages of honey bees. We conclude that the digestive tract of larvae is not sterile or scarcely populated, as reported in earlier studies, but harbours 10^8^ microorganisms primarily from the Firmicutes group. We also found that young bees are inoculated by Firm-5 during a maximum of 6 days post-pupation, and after this time, the counts of Gammaproteobacteria and Firmicutes decline as the honey bee ages.

## Supporting Information

S1 Fig PCR-DGGE profiles of 16S rRNA bacterial genes of all 96 samples. All 96 samples were analysed on multiple gels and were later used for alignment in BioNumerics software. In addition, the gels contain samples of honey and pollen from each experimental hive which were not part of this study.
(PDF)

S2 Fig Phylogenetic tree showing the position of 3 *Lactobacillus* sp. strains examined in this study. Strains are in relation to newly described *Lactobacillus* species isolated from the digestive tract of honey bees (*Apis mellifera*) and bumblebees (*Bombus terrestris*) [23,24,45]. The tree was reconstructed using the maximum-likelihood method based on 16S rRNA (length of 1350 nt) as described previously [46]. Bootstrap values, expressed as percentages of 1000 datasets, are shown at nodes. Numbers in parentheses correspond to GenBank accession numbers. The tree was rooted by *Streptococcus canis* ATCC 43498^T^. Bar, 0.02 substitutions per nucleotide position.
(PDF)

S3 Fig Unrooted phylogenetic tree of representatives of gammaproteobacteria showing the position of 2 bacterial strains discussed in this study. They represent 2 species of recently described new genera within newly established family Orbaceae. Tree was reconstructed using the maximum-likelihood method based on 16S rRNA (length of 1309 nt), as described previously [46]. Bootstrap values, expressed as percentages of 1000 datasets, are shown at nodes. Numbers in parentheses correspond to the GenBank accession numbers. Bar, 0.01 substitutions per nucleotide position. The phylogenetic trees (S2 and S3 Figs.) were viewed using the TreeView (http://taxonomy.zoology.gla.ac.uk/rod/treeview.html) and FigTree (http://tree.bio.ed.ac.uk/software/figtree/) software.
(PDF)

S4 Fig Colony locations map. A, Dol; B, Postrizin; C, Hostice; D, Ustrasice.
(TIFF)

S5 Fig PCR-DGGE profiles of 16S rRNA bacterial genes associated with honey bees during development. Gel shows a mix of honey bee microbial isolates as standard and 2 representative samples of each 1-, 3- and 6-day old larvae (L1, L3 and L6, respectively), white and black pupae (PW, PB), young bees, drones and foraging bees (BY, DR and BF, respectively) used within this study. Bands correspond to *Lactobacillus* spp. (Lac 1–4), *Gilliamella apicola* (Gil 1–6), *Frischella perrara* (Fri), *Bacillus cereus* (Bac), *Snodgrassella alvi* (Sno), *Rhizobiales* bacterium (Rhi) and unknown multiple bands with similarity ˂ 90% (UM). For bands in bold, the V3 region of 16S rRNA sequence was uploaded to NCBI. For others, the highest hit from nBLAST for the DGGE PCR amplicons (∼200 bp) was used for tentative identification.
(PDF)

S1 Table Exp.1 Raw data. Integration data from the band intensities of DGGE gels including 17 bands used for heatmap construction and for RDA analysis.
(XLSX)
