# Supplementary material for: Variation in Honey Bee Gut Microbial Diversity Affected by Ontogenetic Stage, Age and Geographic Location
Source: PLoS One. 2015 Mar 13;10(3):e0118707. doi: 10.1371/journal.pone.0118707 (PMC4358834; doi:10.1371/journal.pone.0118707)
Supplement: S1 Fig — All 96 samples were analysed on multiple gels and were later used for alignment in BioNumerics software. In addition, the gels contain samples of honey and pollen from each experimental hive which were not part of this study. (PDF) [file pone.0118707.s001.pdf]

DGGE profiles of all 96 samples (exp. 1) + samples of honey and pollen

S: standard

L1: 1st instar larvae (1st day)

L3: 4th instar larvae (3rd day)

L6: 5th instar larvae (6th day)

PW: pupae white

PB: pupae black

BY: young bee

BF: foraging bee

DR: drone

HN: honey

PL: pollen

1, 2, 3: hives

A: location, Dol

B: location, Postrizin

C: location, Hostice

D: location, Ustrasice

GEL 1

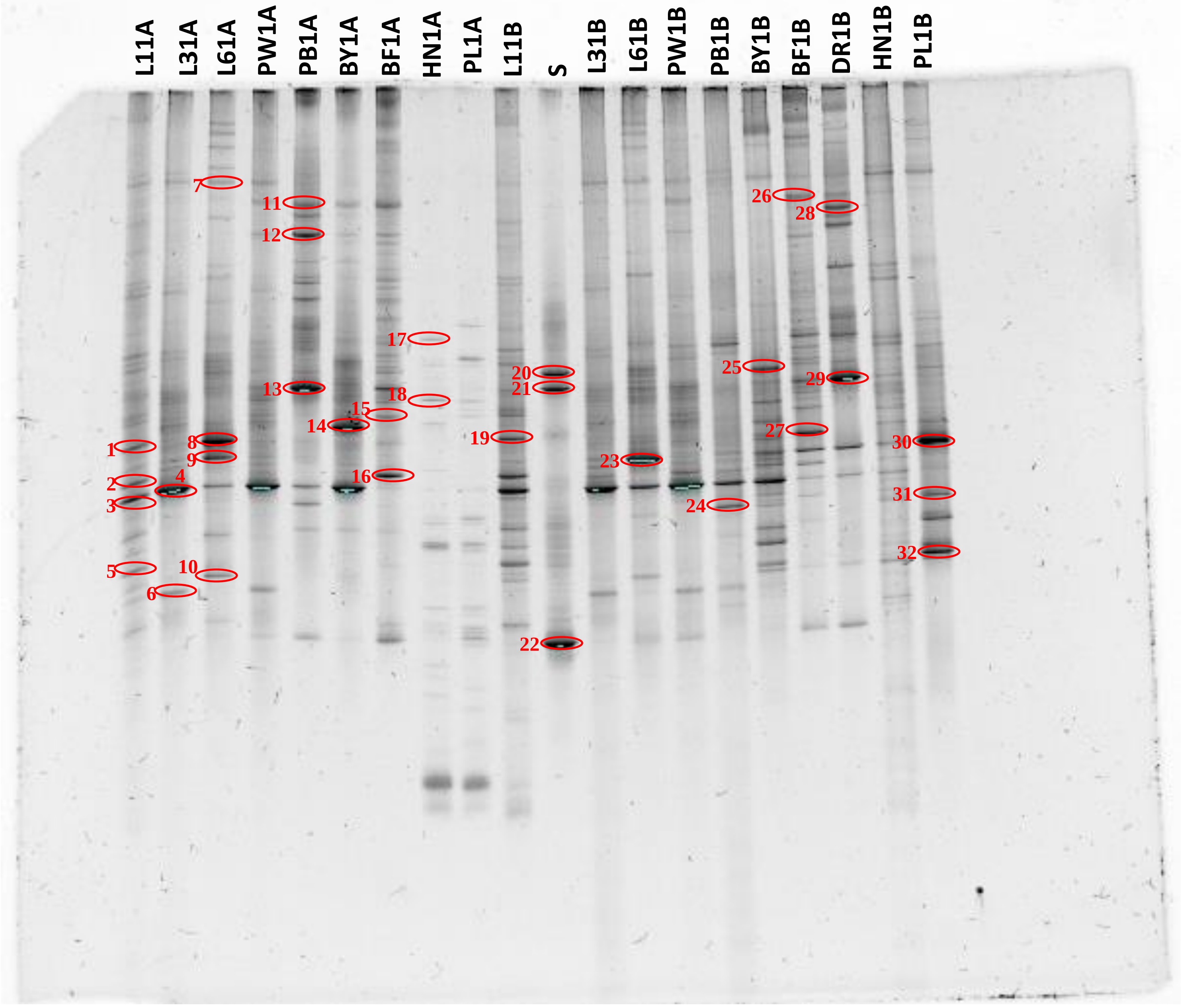
L11A
L31A
L61A
PW1A
PB1A
BY1A
BF1A
HN1A
PL1A
L11B
S
L31B
L61B
PW1B
PB1B
BY1B
BF1B
DR1B
HN1B
PL1B
1
2
3
4
5
6
7
8
9
10
11
12
13
14
15
16
17
18
19
20
21
22
23
24
25
26
27
28
29
30
31
32

GEL 2

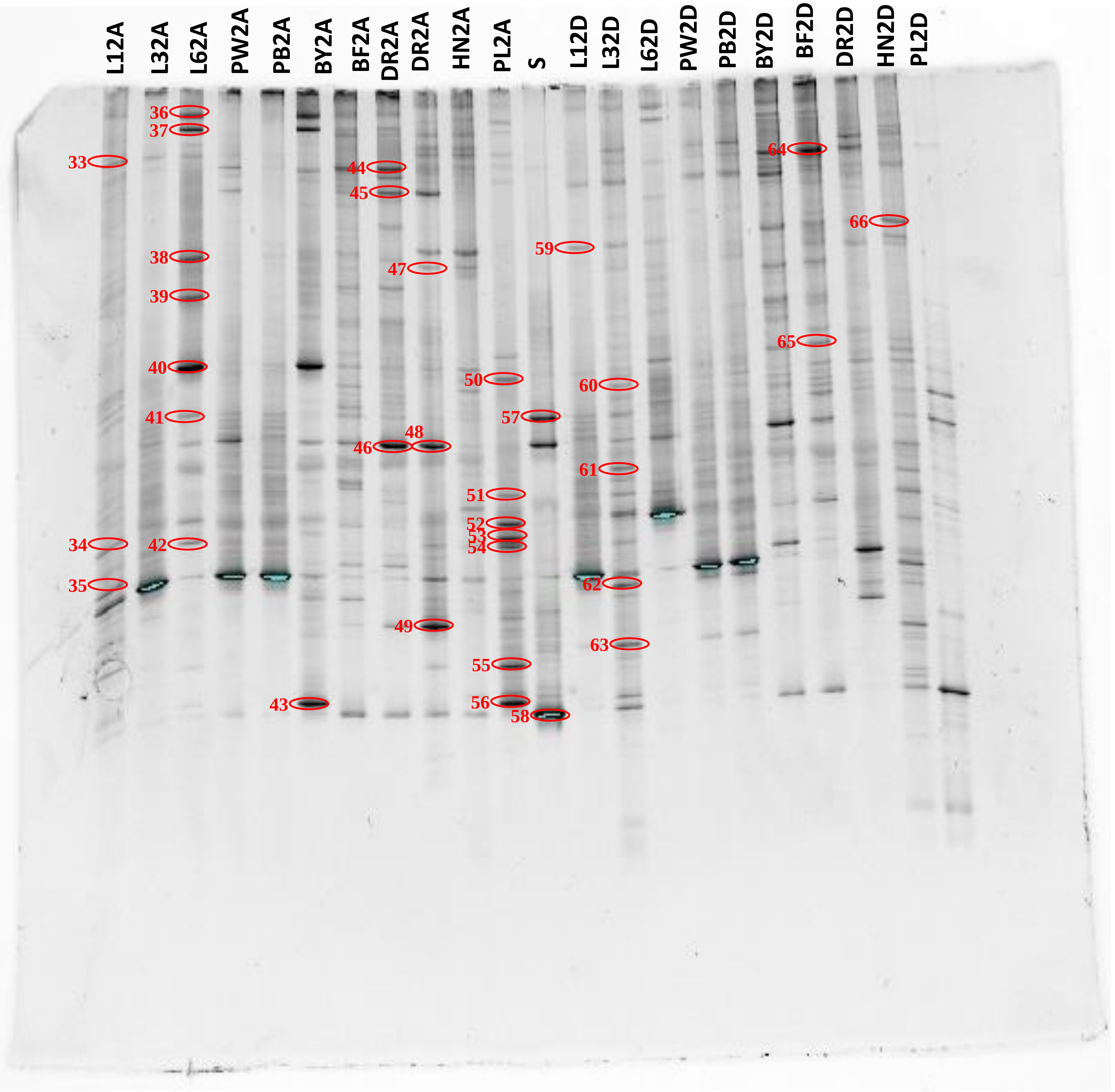
L12A
L32A
L62A
PW2A
PB2A
BY2A
BF2A
DR2A
DR2A
HN2A
PL2A
S
L12D
L32D
L62D
PW2D
PB2D
BY2D
BF2D
DR2D
HN2D
PL2D
33
34
35
36
37
38
39
40
41
42
43
44
45
46
47
48
49
50
51
52
53
54
55
56
57
58
59
60
61
62
63
64
65
66

GEL 3

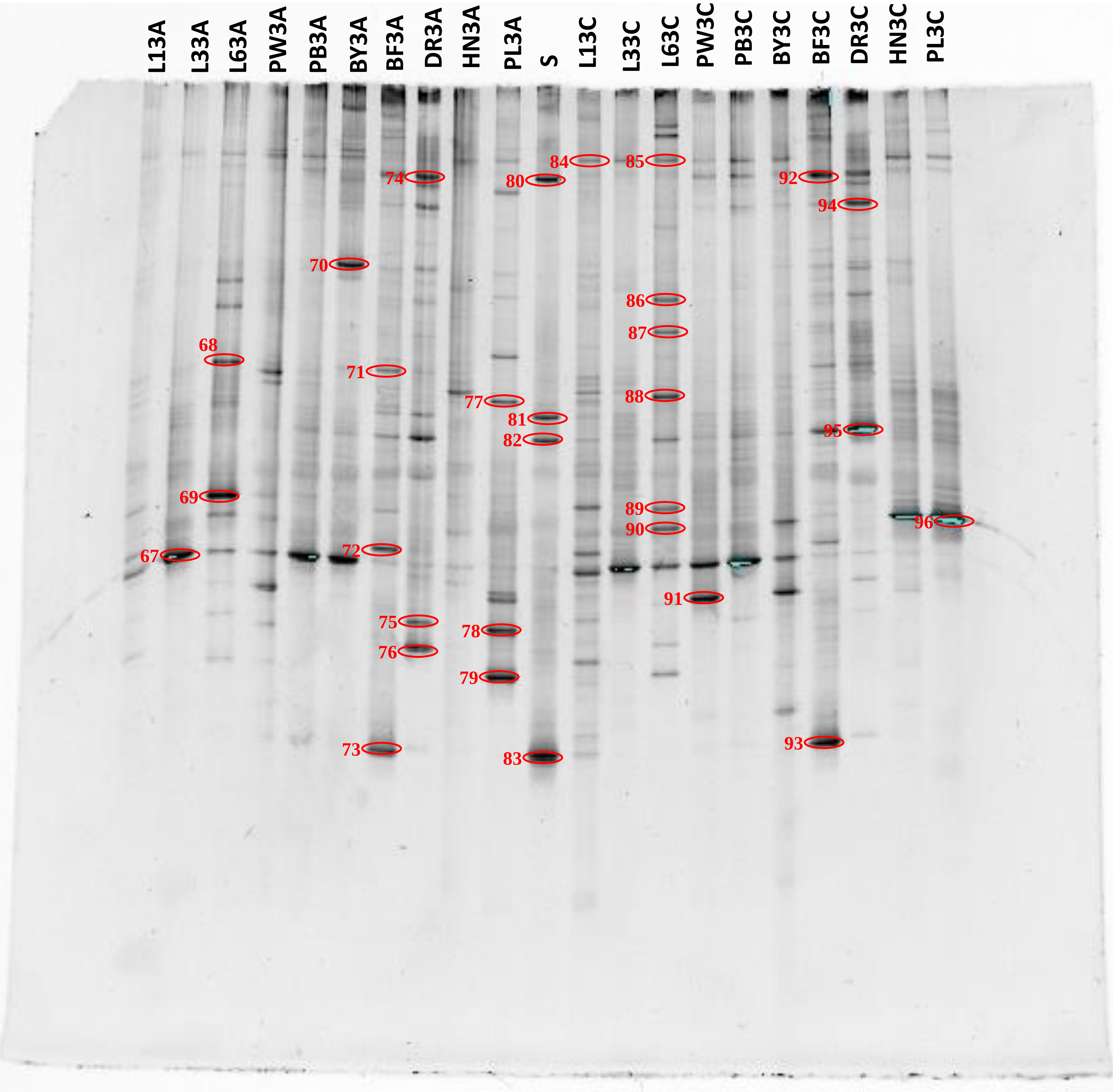
L13A
L33A
L63A
PW3A
PB3A
BY3A
BF3A
DR3A
HN3A
PL3A
S
L13C
L33C
L63C
PW3C
PB3C
BY3C
BF3C
DR3C
HN3C
PL3C
67
68
69
70
71
72
73
74
75
76
77
78
79
80
81
82
83
84
85
86
87
88
89
90
91
92
93
94
95
96

GEL 4

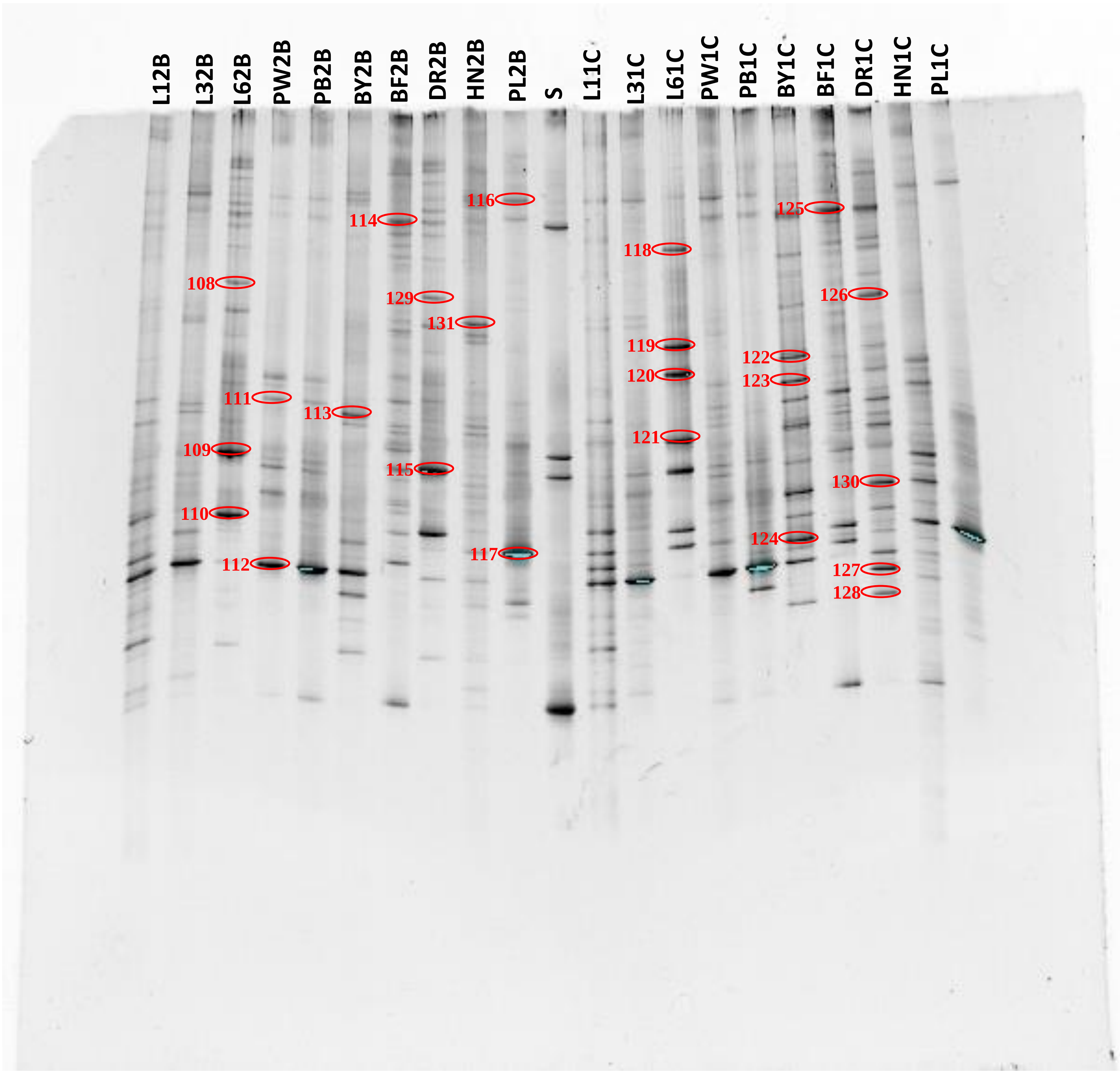

L12B
L32B
L62B
PW2B
PB2B
BY2B
BF2B
DR2B
HN2B
PL2B
S
L11C
L31C
L61C
PW1C
PB1C
BY1C
BF1C
DR1C
HN1C
PL1C
116
114
125
118
108
126
129
131
119
122
120
123
111
113
121
109
115
130
110
124
117
112
127
128

GEL 5

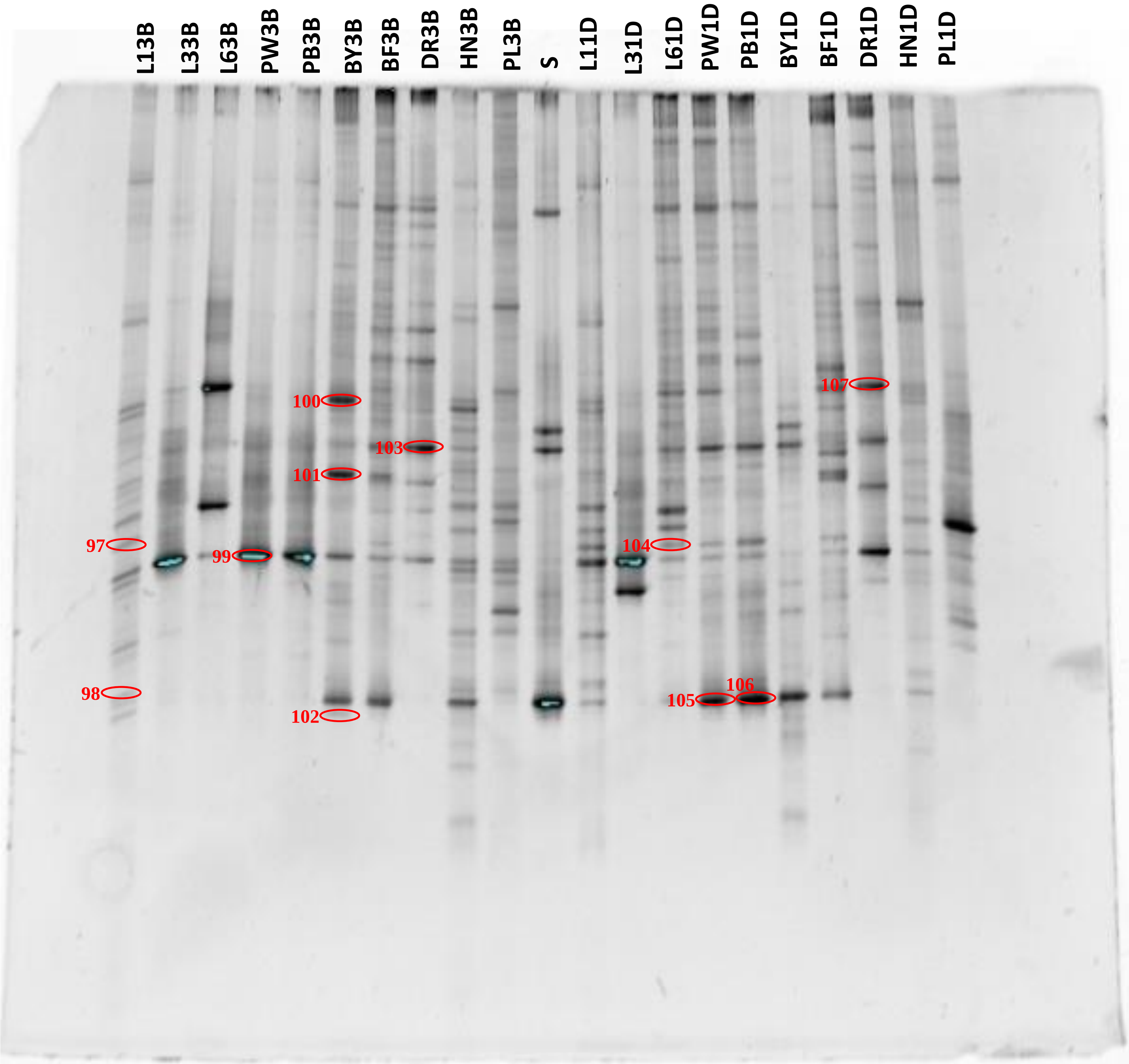
L13B
L33B
L63B
PW3B
PB3B
BY3B
BF3B
DR3B
HN3B
PL3B
S
L11D
L31D
L61D
PW1D
PB1D
BY1D
BF1D
DR1D
HN1D
PL1D
97
98
99
100
101
102
103
104
105
106
107

GEL 6

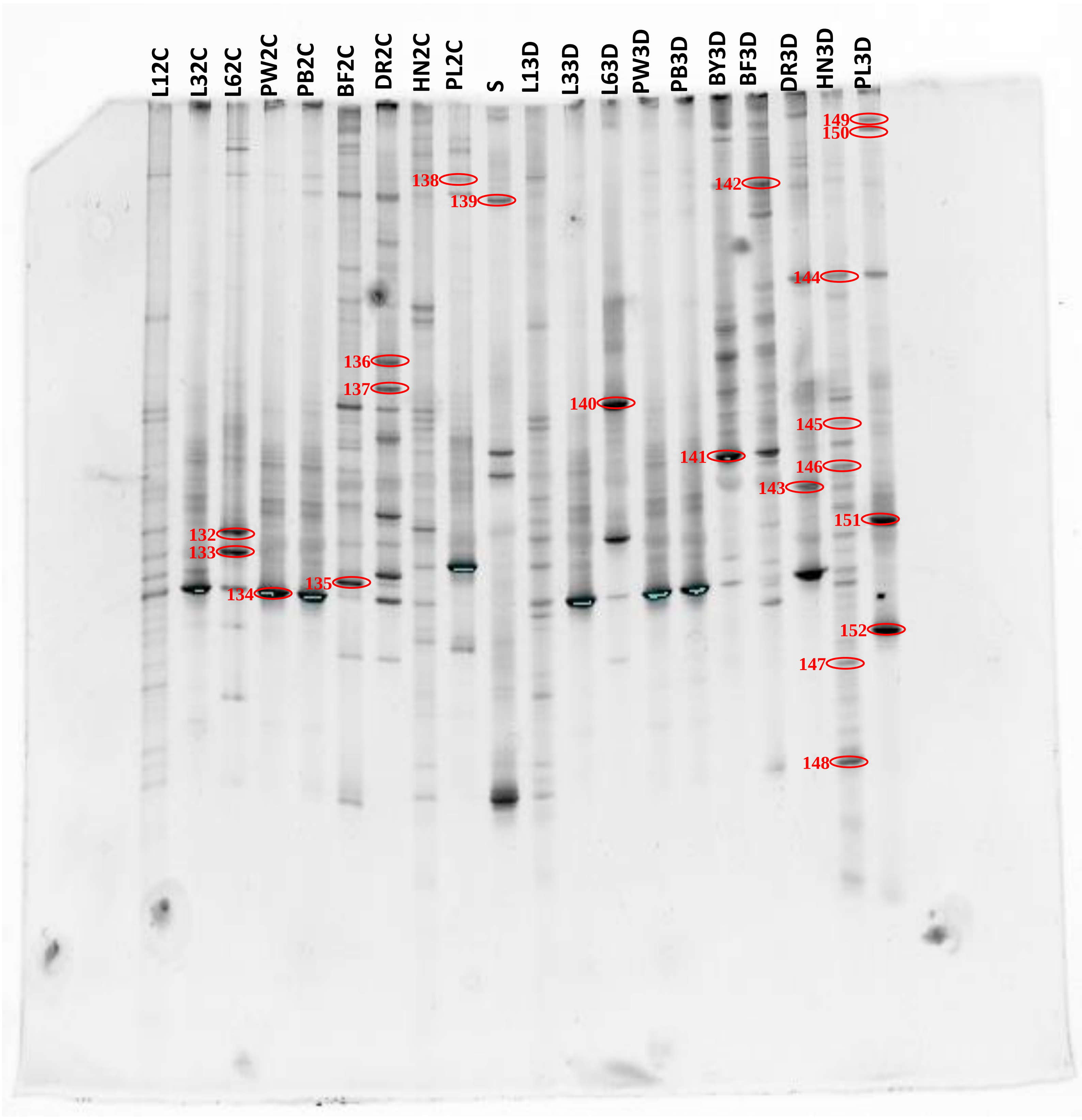
L12C
L32C
L62C
PW2C
PB2C
BF2C
DR2C
HN2C
PL2C
S
L13D
L33D
L63D
PW3D
PB3D
BY3D
BF3D
DR3D
HN3D
PL3D
149
150
138
142
139
144
136
137
140
145
141
146
143
151
132
133
135
134
152
147
148

Band	Identification (%)
**1**	Eukaryotic DNA
**2**	*Snodgrassella alvi* (99)
**3**	Eukaryotic DNA
**4**	Eukaryotic DNA
**5**	*Pseodochrobactrum saccharolyticum* (100)
**6**	*Pseodochrobactrum saccharolyticum* (98)
**7**	unknown multiple bands (<90)
**8**	Eukaryotic DNA
**9**	Eukaryotic DNA
**10**	*Pseodochrobactrum saccharolyticum* (98)
**11**	unknown multiple bands (<90)
**12**	unknown multiple bands (<90)
**13**	*Lactobacillus apis* (100)
**14**	unknown multiple bands (<90)
**15**	unknown multiple bands (<90)
**16**	*Snodgrassella alvi* (99)
**17**	*Pasteurella* (99)
**18**	*Pseudomonadales* (93)
**19**	Eukaryotic DNA
**20**	*Lactobacillus melliventris* (99)
**21**	*Lactobacillus apis* (99)
**22**	*Bifidobacterium asteroides* (100)
**23**	Eukaryotic DNA
**24**	Eukaryotic DNA
**25**	*Klebsiella oxytoca* (99)
**26**	unknown multiple bands (<90)
**27**	Eukaryotic DNA

Band	Identification (%)
**28**	*Lactobacillus apis* (98)
**29**	*Lactobacillus* sp. (99)
**30**	Eukaryotic DNA
**31**	*Pseodochrobactrum saccharolyticum* (100)
**32**	*Pseodochrobactrum saccharolyticum* (100)
**33**	*Lactobacillus apis* (99)
**34**	Eukaryotic DNA
**35**	Eukaryotic DNA
**36**	*Gilliamella apicola* (97)
**37**	unknown multiple bands (<90)
**38**	*Frischella perrara* (100)
**39**	*Frischella perrara* (100)
**40**	*Frischella perrara* (100)
**41**	*Lactobacillus melliventris* (99)
**42**	Eukaryotic DNA
**43**	*Bifidobacterium asteroides* (100)
**44**	*Lactobacillus apis* (99)
**45**	*Lactobacillus apis* (98)
**46**	unknown multiple bands (<90)
**47**	*Frischella perrara* (98)
**48**	unknown multiple bands (<90)
**49**	*Pseodochrobactrum saccharolyticum* (99)
**50**	unknown multiple bands (<90)
**51**	unknown multiple bands (<90)
**52**	unknown multiple bands (<90)
**53**	Eukaryotic DNA
**54**	Eukaryotic DNA

Band	Identification (%)
**55**	unknown multiple bands (<90)
**56**	*Bifidobacterium asteroides* (99)
**57**	*Lactobacillus melliventris* (99)
**58**	*Bifidobacterium asteroides* (100)
**59**	*Frischella perrara* (100)
**60**	unknown multiple bands (<90)
**61**	unknown multiple bands (<90)
**62**	Eukaryotic DNA
**63**	unknown multiple bands (<90)
**64**	*Lactobacillus apis* (97)
**65**	unknown multiple bands (<90)
**66**	*Frischella perrara* (100)
**67**	Eukaryotic DNA
**68**	*Frischella perrara* (100)
**69**	unknown multiple bands (<90)
**70**	*Frischella perrara* (99)
**71**	unknown multiple bands (<90)
**72**	unknown multiple bands (<90)
**73**	*Rhizobiales* bacterium (100)
**74**	*Lactobacillus apis* (97)
**75**	*Pseodochrobactrum saccharolyticum* (100)
**76**	*Carnimonas nigrificans* (96)
**77**	Uncultured Cyanobacterium (99)
**78**	*Pseodochrobactrum saccharolyticum* (97)
**79**	*Halospirulina* sp. (100)
**80**	*Lactobacillus apis* (99)
**81**	*Lactobacillus melliventris* (99)

Band	Identification (%)
**82**	*Lactobacillus apis* (99)
**83**	*Bifidobacterium asteroides* (100)
**84**	unknown multiple bands (<90)
**85**	unknown multiple bands (<90)
**86**	*Gilliamella apicola* (100)
**87**	*Gilliamella apicola* (100)
**88**	*Gilliamella apicola* (100)
**89**	Eukaryotic DNA
**90**	Eukaryotic DNA
**91**	Eukaryotic DNA
**92**	*Lactobacillus melliventris* (95)
**93**	*Rhizobiales* bacterium (100)
**94**	*Lactobacillus* sp. (99)
**95**	*Lactobacillus* sp. (99)
**96**	Eukaryotic DNA
**97**	Eukaryotic DNA
**98**	Eukaryotic DNA
**99**	Eukaryotic DNA
**100**	*Spiroplasma apis* (95)
**101**	Uncultured betaproteobacterium (98)
**102**	unknown multiple bands (<90)
**103**	*Lactobacillus melliventris* (99)
**104**	*Snodgrassella alvi* (100)
**105**	*Pseudoochrobactrum saccharolyticum* (100)
**106**	*Pseudoochrobactrum saccharolyticum* (100)
**107**	*Frischella perrara* (100)
**108**	*Gilliamella apicola* (94)

**Band**	**Identification (%)**
**109**	*Gilliamella apicola* (95)
**110**	unknown multiple bands (<90)
**111**	unknown multiple bands (<90)
**112**	Eukaryotic DNA
**113**	unknown multiple bands (<90)
**114**	*Lactobacillus* sp. (94)
**115**	unknown multiple bands (<90)
**116**	unknown multiple bands (<90)
**117**	*Snodgrassella alvi* (99)
**118**	*Lactobacillus apis* (99)
**119**	*Gilliamella apicola* (99)
**120**	*Gilliamella apicola* (97)
**121**	*Lactobacillus melliventris* (100)
**122**	*Gilliamella apicola* (99)
**123**	*Gilliamella apicola* (99)
**124**	unknown multiple bands (<90)
**125**	*Lactobacillus* sp. (99)
**126**	*Frischella perrara* (100)
**127**	Eukaryotic DNA
**128**	unknown multiple bands (<90)
**129**	*Frischella perrara* (97)
**130**	unknown multiple bands (<90)
**131**	*Frischella perrara* (96)
**132**	Eukaryotic DNA
**133**	Eukaryotic DNA
**134**	Eukaryotic DNA
**135**	Snodgrassella alvi (99)

**Band**	**Identification (%)**
**136**	*Lactobacillus* sp. (99)
**137**	*Lactobacillus* sp. (99)
**138**	unknown multiple bands (<90)
**139**	*Lactobacillus apis* (99)
**140**	unknown multiple bands (<90)
**141**	*Lactobacillus apis* (99)
**142**	*Lactobacillus* sp. (93)
**143**	unknown multiple bands (<90)
**144**	*Frischella perrara* (99)
**145**	*Lactobacillus melliventris* (99)
**146**	unknown multiple bands (<90)
**147**	*Pseodochrobactrum saccharolyticum* (100)
**148**	*Bifidobacterium asteroides* (98)
**149**	unknown multiple bands (<90)
**150**	unknown multiple bands (<90)
**151**	uncultured bacterium (99)
**152**	uncultured bacterium (99)
