# Supplementary figures and images for: Variation in Honey Bee Gut Microbial Diversity Affected by Ontogenetic Stage, Age and Geographic Location
Source: PLoS One. 2015 Mar 13;10(3):e0118707. doi: 10.1371/journal.pone.0118707 (PMC4358834; doi:10.1371/journal.pone.0118707)

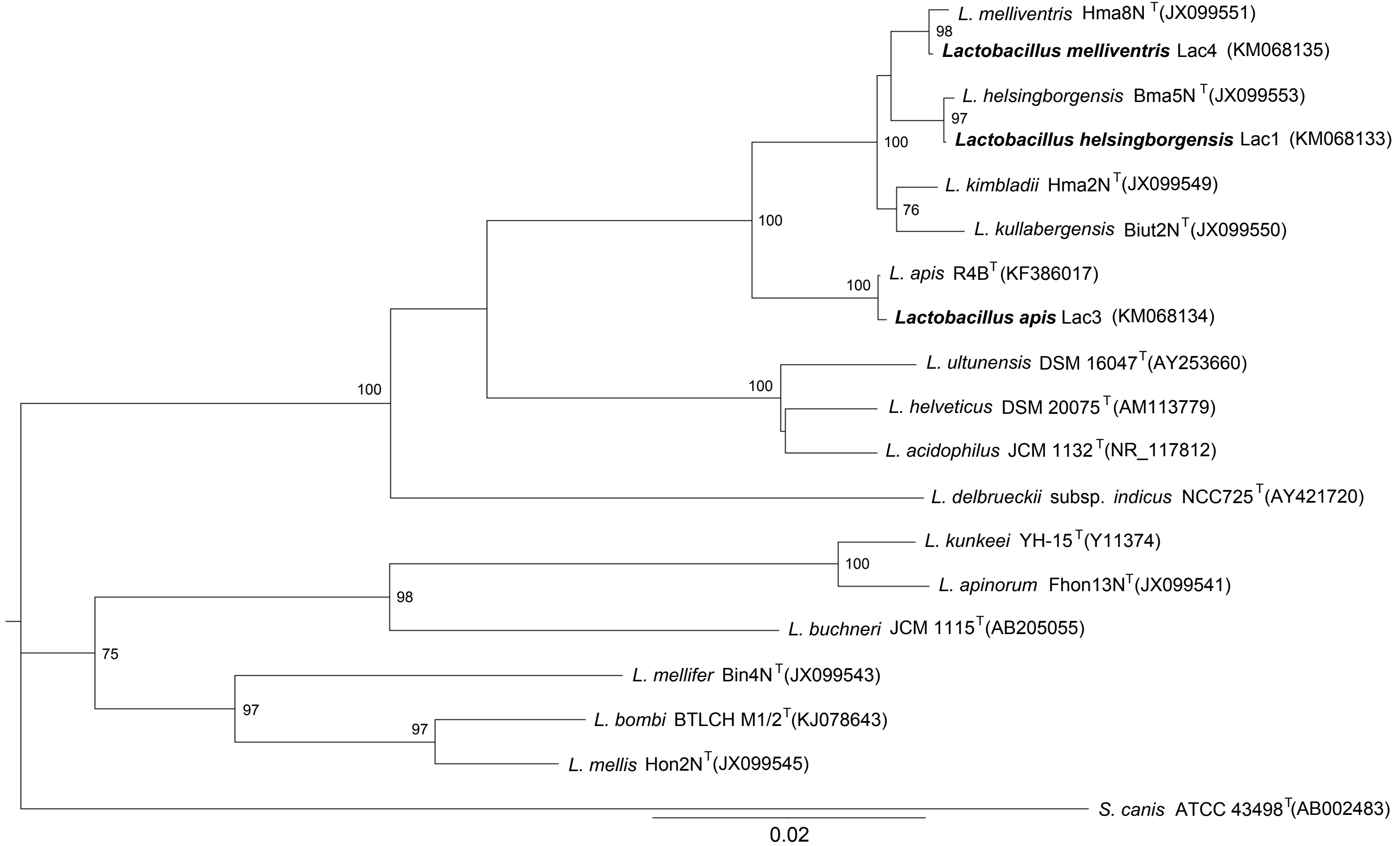

Supplement: S2 Fig — Strains are in relation to newly described Lactobacillus species isolated from the digestive tract of honey bees (Apis mellifera) and bumblebees (Bombus terrestris) [23,24,45]. The tree was reconstructed using the maximum-likelihood method based on 16S rRNA (length of 1350 nt) as described previously [46]. Bootstrap values, expressed as percentages of 1000 datasets, are shown at nodes. Numbers in parentheses correspond to GenBank accession numbers. The tree was rooted by Streptococcus canis ATCC 43498T. Bar, 0.02 substitutions per nucleotide position. (PDF) [file pone.0118707.s002.pdf]

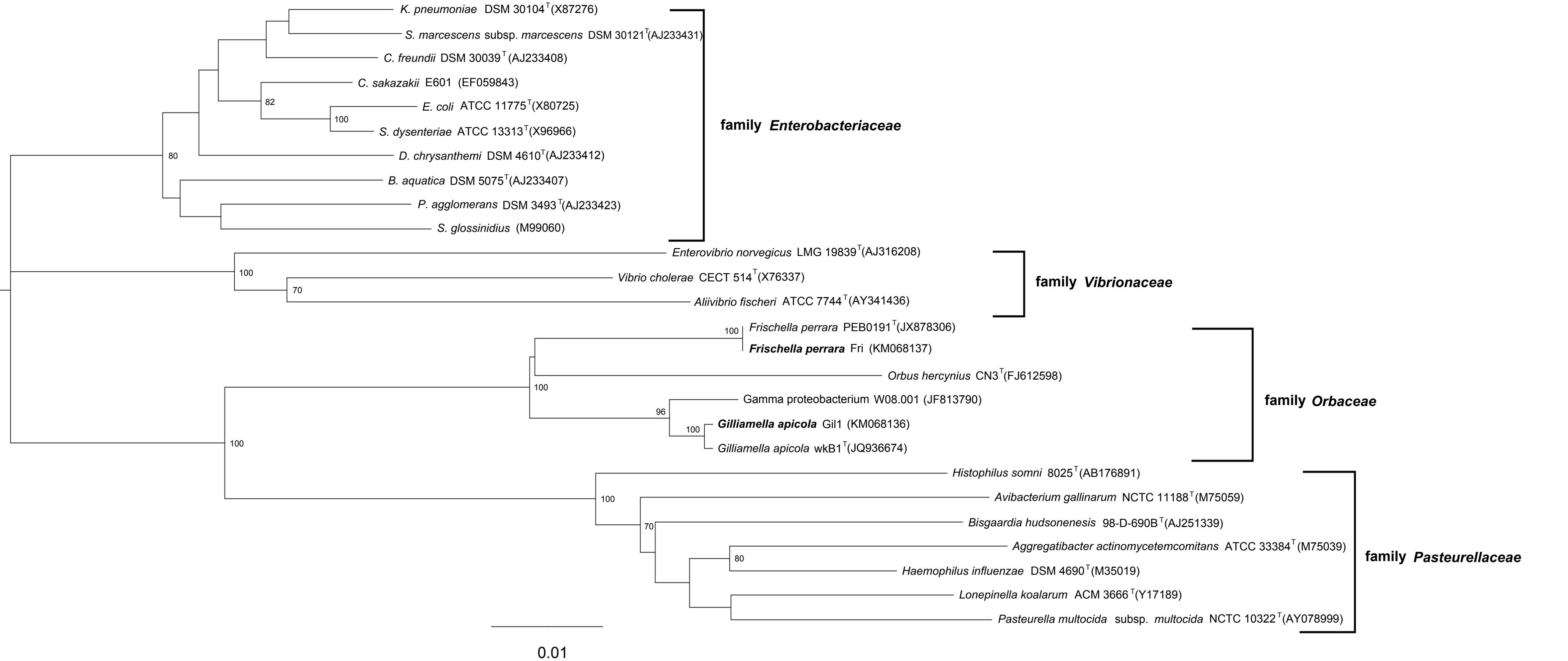

Supplement: S3 Fig — They represent 2 species of recently described new genera within newly established family Orbaceae. Tree was reconstructed using the maximum-likelihood method based on 16S rRNA (length of 1309 nt), as described previously [46]. Bootstrap values, expressed as percentages of 1000 datasets, are shown at nodes. Numbers in parentheses correspond to the GenBank accession numbers. Bar, 0.01 substitutions per nucleotide position. The phylogenetic trees (S2 and S3 Figs.) were viewed using the TreeView (http://taxonomy.zoology.gla.ac.uk/rod/treeview.html) and FigTree (http://tree.bio.ed.ac.uk/software/figtree/) software. (PDF) [file pone.0118707.s003.pdf]

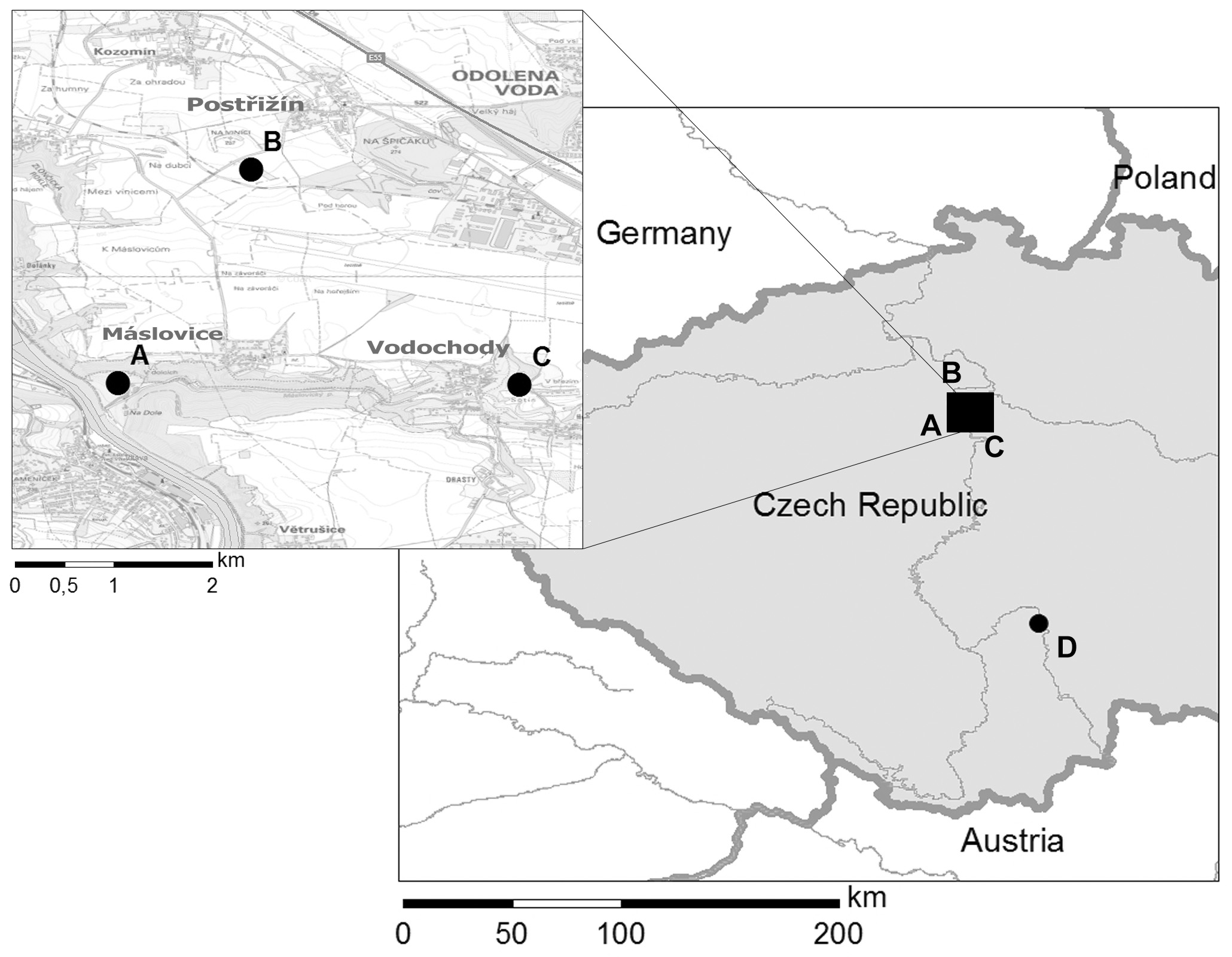

Supplement: S4 Fig — A, Dol; B, Postrizin; C, Hostice; D, Ustrasice. (TIFF) [file pone.0118707.s004.tiff]

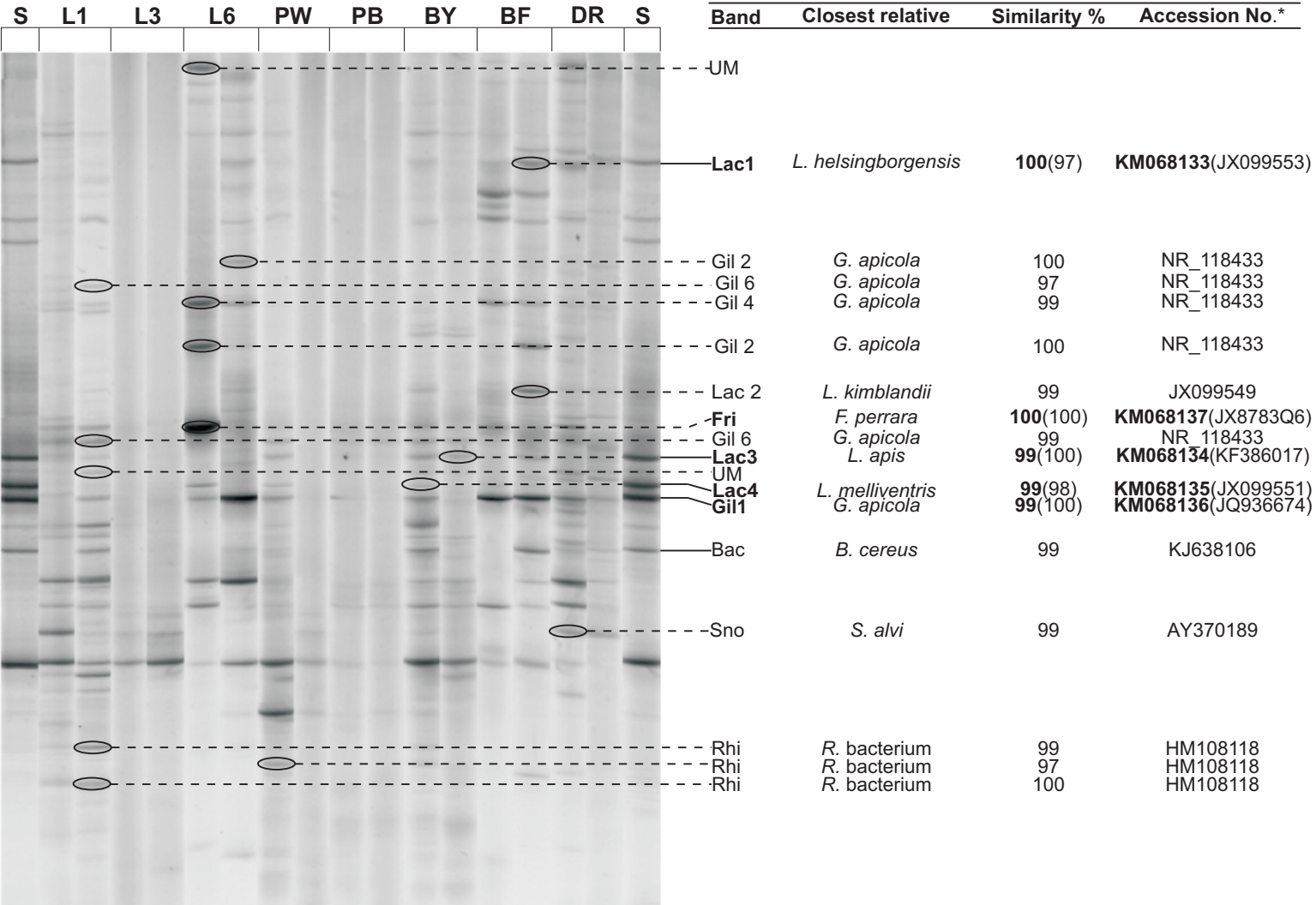

Supplement: S5 Fig — Gel shows a mix of honey bee microbial isolates as standard and 2 representative samples of each 1-, 3- and 6-day old larvae (L1, L3 and L6, respectively), white and black pupae (PW, PB), young bees, drones and foraging bees (BY, DR and BF, respectively) used within this study. Bands correspond to Lactobacillus spp. (Lac 1–4), Gilliamella apicola (Gil 1–6), Frischella perrara (Fri), Bacillus cereus (Bac), Snodgrassella alvi (Sno), Rhizobiales bacterium (Rhi) and unknown multiple bands with similarity ˂ 90% (UM). For bands in bold, the V3 region of 16S rRNA sequence was uploaded to NCBI. For others, the highest hit from nBLAST for the DGGE PCR amplicons (∼200 bp) was used for tentative identification. (PDF) [file pone.0118707.s005.pdf]
